# Technical validation of a multimodal emotion-adaptive biofeedback system for autonomic regulation using guided breathing

**DOI:** 10.1038/s41598-026-46105-9

**Published:** 2026-04-01

**Authors:** C. R. Srinivasan, Pulkit Kumar, S. Meenatchi Sundaram

**Affiliations:** Manipal Institute of Technology, Manipal Academy of Higher Education, Manipal, India

**Keywords:** Multimodal biofeedback, Resonance breathing, Adaptive algorithms, Emotion recognition, Autonomic modulation, Digital health, Closed-loop biofeedback, Computational biology and bioinformatics, Engineering, Health care, Physiology

## Abstract

Breathing interventions delivered through biofeedback have been shown to support physiological modulation consistent with resonance breathing responses. However, most biofeedback systems on the market are based on fixed procedures, provide limited physiological feedback, and do not adapt to user comfort or emotional state. The system is designed to work under a typical 20-min biofeedback timeframe and uses a two-stage adaptive respiration rate (RR) algorithm that gradually converges toward resonance frequency while dynamically responding to signs of emotional discomfort. System-level testing was conducted using reference-grade measurement instruments. Respiration rate estimation showed low mean absolute error, while heart rate and heart rate variability measures demonstrated high agreement with reference standards. During adaptive biofeedback, the system was associated with a decrease in respiratory rate and electrodermal activity, along with increased heart rate variability, consistent with increased parasympathetic influence inferred from HRV trends. These results indicate the technical validity and system-level feasibility of an emotion-aware multimodal biofeedback framework. The proposed framework provides a scalable foundation for future controlled and longitudinal validation studies.

**Supplementary Information:**

The online version contains supplementary material available at 10.1038/s41598-026-46105-9.

## Introduction

Biofeedback (BF) is a non-invasive form of autonomic nervous system (ANS) modulation that has been extensively studied in the context of allowing the individual to have voluntary control of physiological mechanisms, including the respiration of the body system, heart rate and electrodermal activity^1–4^. Heart rate variability biofeedback (HRV-BF) and respiratory biofeedback (RBF) among others have proven to be relevant to stress management and psychophysiological health by stimulation of parasympathetic activity via paced or resonance-frequency breathing^5–7^.

The biophysiological explanation of the HRV- and respiration-based biofeedback is that cardiac and respiratory rhythm are closely linked. The changes in respiration adjust the heart rate in respiratory sinus arrhythmia, which indicates the dynamic relationship between sympathetic and parasympathetic sections of the ANS^7^. Breathing at a rate close to the resonance frequency of the person, usually in the range of 4.5 to 6 breaths per minute, has been demonstrated to achieve the highest possible HRV amplitude and enhance autonomic control^8,9^. Notably, the HRV measures were also linked to affective states, where positive emotions were linked to a high HRV coherence and negative emotions were associated with sympathetic dominance^10^. This evidence contributes to the importance of the emotional context in biofeedback feasibility.

Irrespective of this robust physiological premise, most of the currently available biofeedback systems are still weak in terms of providing adaptive and user-focused interventions. Commercially available wearable devices, which measure respiration and cardiovascular parameters based on photoplethysmography (PPG) and inertial sensors, also have limitations to their reliability and availability due to accuracy, cost, and requiring skin contact problems. Correspondingly, the most common mobile guides breathing applications offer a static visual- or audio-based pacing but do not include closed-loop physiological feedback and real-time personalization, greatly depending on user compliance^11,12^.

The latest developments in sensing and computation have made it possible to adopt alternative methods, such as camera-based respiration estimation with convolutional neural networks, which have proved to be promisingly accurate in a controlled setting^13^. However, these techniques are still vulnerable to the environmental conditions of lighting, posture and movement and may be less robust in practical settings. In a broader sense, earlier studies on biofeedback have mainly focused on the evaluation of system-level outcomes through commercial packages instead of the underlying system design issues, such as multimodal data collection, real time adjustment, and comfort of users in the course of the intervention^12,14,15^.

**Fig. 1:**
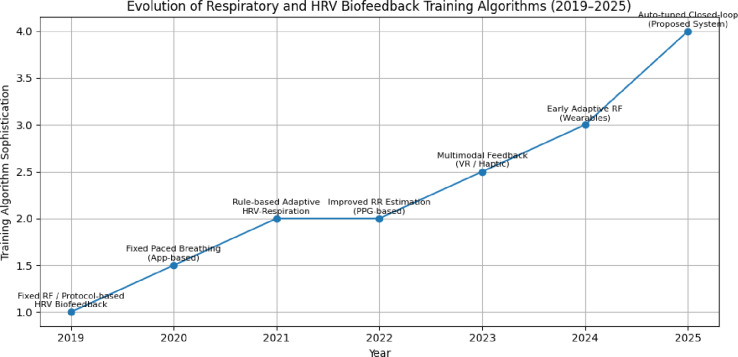
Evolution of biofeedback algorithms from 2019–2025.

According to the summary in Fig. 1, the majority of modern biofeedback devices use fixed or protocol-driven breathing instructions, typically based on a preset resonance frequency (e.g. 0.1 Hz), and with little intra-session variability. Although more recent methods have proposed automatic tuning, these methods are usually single objective, rule-based and optimize only physiological metrics like HRV coherence^1,2,7^. The existing biofeedback systems therefore do not have the necessary emotion-sensitive, multimodal dynamism and cannot therefore react to dynamic changes in physiological and emotional responses in a real-life setting.

The key contributions of this work are threefold. First, we propose a multimodal biofeedback architecture integrating respiration, ECG, electrodermal activity, and real-time facial emotion inference within a unified closed-loop framework. Second, we introduce a two-stage emotion-aware adaptive respiration algorithm that dynamically adjusts breathing toward resonance frequency while preserving user comfort through affective-state modulation. Third, we provide system-level technical validation against reference-grade instruments with synchronized multimodal agreement analysis. Unlike prior HRV or resonance breathing biofeedback systems that rely primarily on fixed or physiology-only adaptation, the proposed framework incorporates emotion-sensitive adaptive control and cloud-assisted real-time processing, establishing a foundation for next-generation personalized biofeedback systems. Emotion inference is used solely as a contextual adaptive input and not as a clinical affective diagnostic tool.

Despite advances in wearable sensing and biofeedback technologies, most existing systems remain limited by fixed breathing protocols, lack of multimodal integration, and minimal responsiveness to user comfort or emotional state. Addressing these gaps is essential for translating laboratory-based biofeedback protocols into scalable personalized digital health interventions suitable for real-world deployment.

In response to these limitations, this study presents the design and technical validation of a multimodal emotion-adaptive biofeedback system integrating respiration, electrocardiography (ECG), electrodermal activity, and real-time facial emotion recognition^13,16–19^. The proposed framework is designed to support physiological modulation and enhance user comfort through emotion-aware adaptive respiration control^20–22^.

## System hardware architecture and signal processing

### Data acquisition and preprocessing

#### Respiration sensing and processing

The respiratory activity was recorded with an MPU6050 inertial measurement unit (IMU) and the gyroscope y-axis was set to measure an angular velocity in the sagittal plane which represented anteroposterior motion of the thoracic area during breathing^23–26^. A low-pass filter was also turned on (DLPF) whose cutoff frequency was 21 Hz to remove high-frequency noise and mechanical vibrations to enhance the quality of the signal. The full-scale range of about ± 250°/s was sensitive enough to record minute respiratory movement and not saturated during impulsive postural changes in the body^24^.

**Fig. 2:**
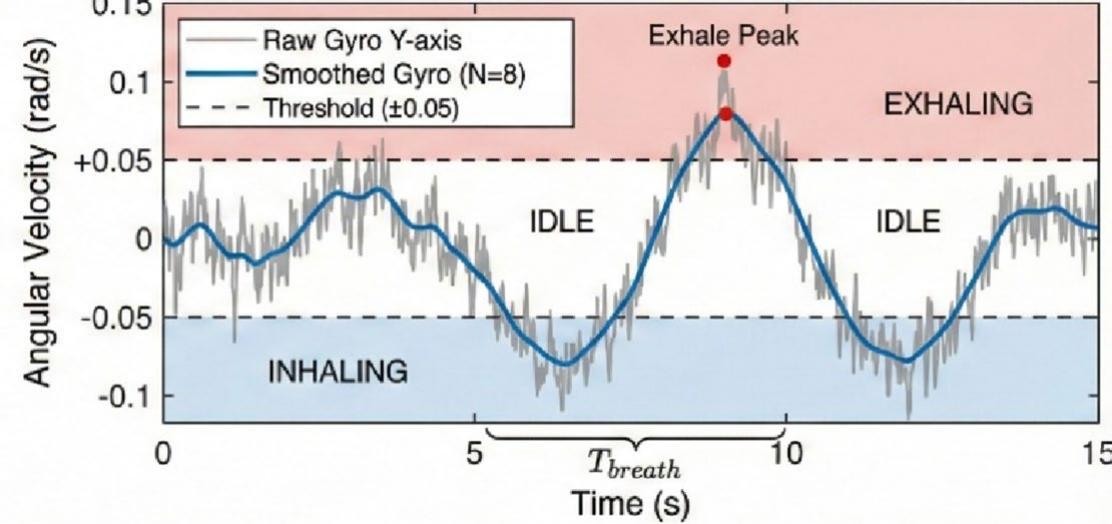
Real-time respiration phase detection via MPU6050 gyroscope data showing a valid breath of approximately 5000 ms.

Digital samples of the angular velocity were read at a frequency of 50 Hz at a 12-bit analog-to-digital converter and real-time processed in three consecutive stages on an ESP32 microcontroller^13,17–19^ (Fig. 2).

Moving average filter A moving average filter was used with window size of M = 8 by filtering a circular buffer; high-frequency variations were minimized, but morphology of respiratory waveforms was retained:

1 $${\omega _{{\mathrm{smooth}}}}\left( n \right)=\frac{1}{M}\sum\limits_{{i=0}}^{{M - 1}} {{S_y}\left( {n - i} \right)}$$

where ${\omega}_{\mathrm{smooth}}\left(n\right)$ is the smoothed angular velocity at time *n*, ${S}_{y}(n-i)$ is the raw gyroscope sample, and *M* is the buffer length.

,Respiratory phase detection was carried out as a three-state finite state machine which includes an Idle, Inhale and Exhale state. Phase changes were determined by crossing the workshear signal (− 0.05 rad/s, + 0.05 rad/s and + 0.01 rad/s, respectively) threshold crossings, and a time stamp was taken at every transition point 3. Breath validity was determined by ensuring that the total cycle was between 500 ms and 15 s to eliminate artifacts due to speech, coughing, or motion^25,32–35^.

Valid breaths yielded inhalation and exhalation durations, from which the inhalation–exhalation (I/E) ratio was computed:

2 $${\mathrm{I:E~Ratio}}=\frac{{{\Delta}{t_{{{inhale}}}}}}{{{\Delta}{t_{{\mathrm{exhale}}}}}}$$

Respiration rate (RR) was estimated using a rolling average over the 15 most recent valid breaths stored in a circular buffer^9^:

3 $${\mathrm{RR}}({\mathrm{BPM}})=\frac{{{N_{{\mathrm{breaths}}}}}}{{\Delta t}} \times 60$$

where ${N}_{\mathrm{breaths}}$ denotes the number of breaths within the buffer and ${\Delta} t$ is the elapsed time in seconds.

#### Electrocardiogram acquisition and heart rate analysis

The electrocardiographic data were recorded with the AD8232 analog front-end, which was chosen due to its large common-mode rejection ratio (> 80 dB), low power usage and applicability to ambulatory monitoring. A common three electrode Lead-I set up comprising Ag/AgCl electrodes was used. Signal amplification, high-pass filtering to remove baseline wander and low-pass filtering to eliminate electromyographic and power-line noise were done by the front-end^26–29^.

**Fig. 3:**
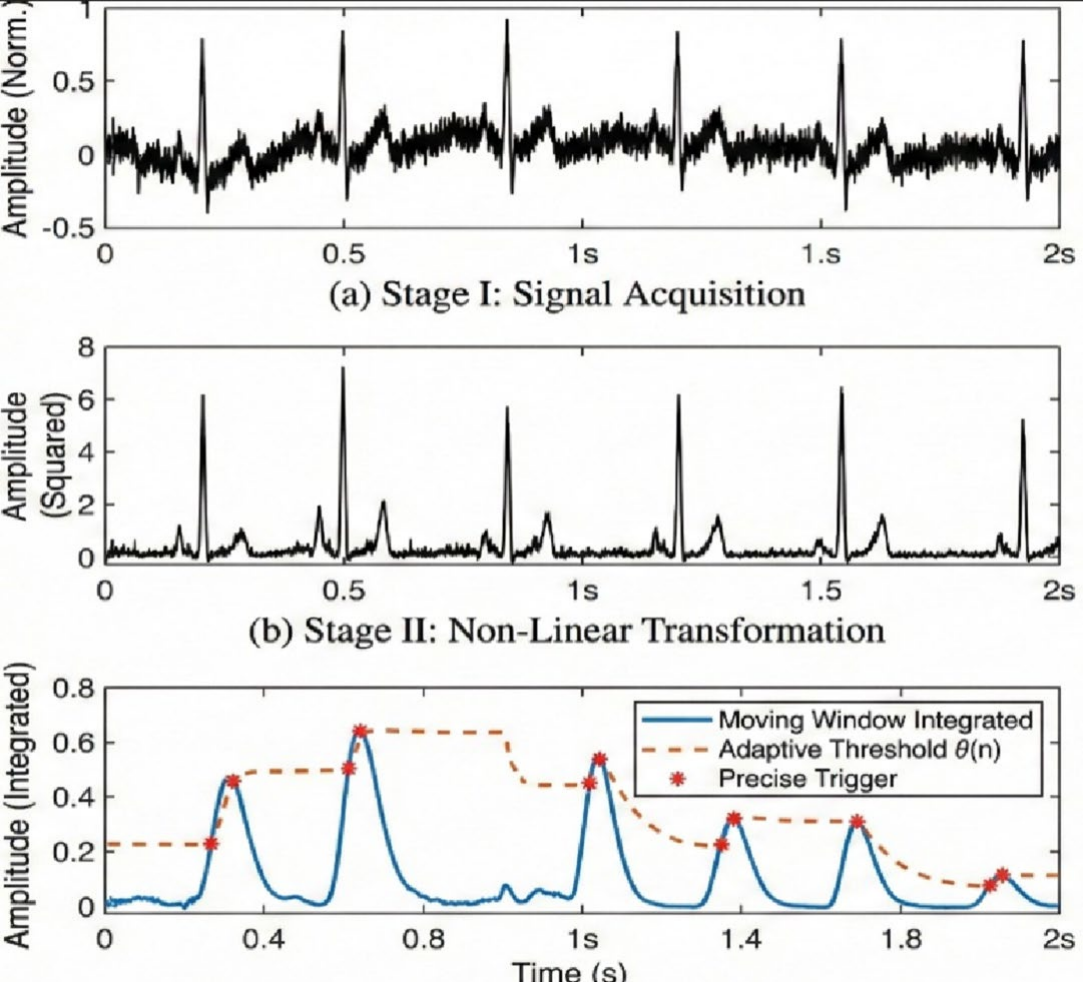
Multistage Pan-Tompkins QRS detection algorithm.

The ESP32 12-bit ADC digitized conditioned ECG signals at 250 Hz, which is sufficiently high to allow to detect R-peaks and compute HRV. The R-peaks were detected by a real-time application of the Pan-Tompkins algorithm which incorporates bandpass filtering (5–15 Hz), differentiation, signal-squaring, and moving-window integration with adaptive-thresholding after the moving-window integration (Fig. 3).

After detection of R-peaks, interbeat (NN) intervals were calculated in milliseconds, and physiologically believable interbeat (NN) intervals relative to heart rates below 40 and above 200 BPM were eliminated 8. Heart rate was derived as:

4 $${\text{HR (BPM)}}=\frac{{60{\mathrm{,}}000}}{{{\mathrm{N}}{{\mathrm{N}}_i}}}$$

where ${\mathrm{NN}}_{i}$ is the length of normal interval of the heartbeat number i. This produced clean NN series upon which standard HRV measures applicable to biofeedback were computed.

#### Electrodermal activity acquisition and trend analysis

The electrodermal activity (EDA) was recorded with a two-electrode bipolar setup to record the changes in skin conductance that are inversely proportional to skin impedance^19,43 –52^. A 12-bit ADC was used to digitize signals at 10 Hz and this sampling rate was adequate to record rapid phasic skin conductance responses (SCRs) as well as slower tonic skin conductance levels (SCLs^13^).

**Fig. 4:**
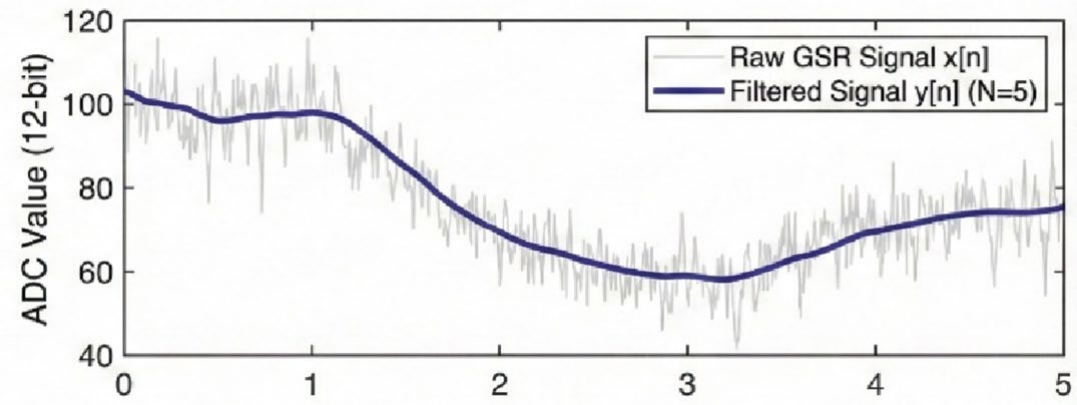
Raw and filtered signals of the GSR response.

A moving average filter (*N* = 5) was used to reduce high-frequency noise and other small artifacts in the raw EDA signal (Fig. 4). The filtered signal was further processed to determine tonic and phasic signals. An analysis of trend module calculated the first derivative of the smoothed signal with a sliding window to give an approximation of the dynamics of sympathetic arousal in real-time. Positive derivatives signified the reaction of growing arousal, and negative derivatives signified recovery or relaxation^26–37^. Such trend estimate was one of the main inputs to the adaptive biofeedback control logic.

### System software architecture and computational framework

A scalable software architecture incorporating cloud assistance was implemented to facilitate real-time physiological monitoring, computationally intensive analytics, and secure long-term data storage. The three-layered system pipeline includes edge-level acquisition, server-side computing, and client-level visualization, which guarantees signal processing of high fidelity, low-latency feedback, and data integrity throughout the biofeedback sessions.

#### Edge-level processing and data streaming

At the edge layer, a wearable platform based on an ESP32 microcontroller records multimodal physiological data, such as ECG, respiration, and electrodermal activity, and includes low-latency preprocessing that can receive primary physiological variables (respiration cycles, NN/R -R intervals, heart rate, and electrodermal trends). Such metrics are converted into lightweight JSON-encoded packets and transmitted continuously to the server over a persistent WebSocket connection to incur minimum communication overhead and have time-synchronized information.

#### Multimodal signal processing pipeline

**Fig. 5:**
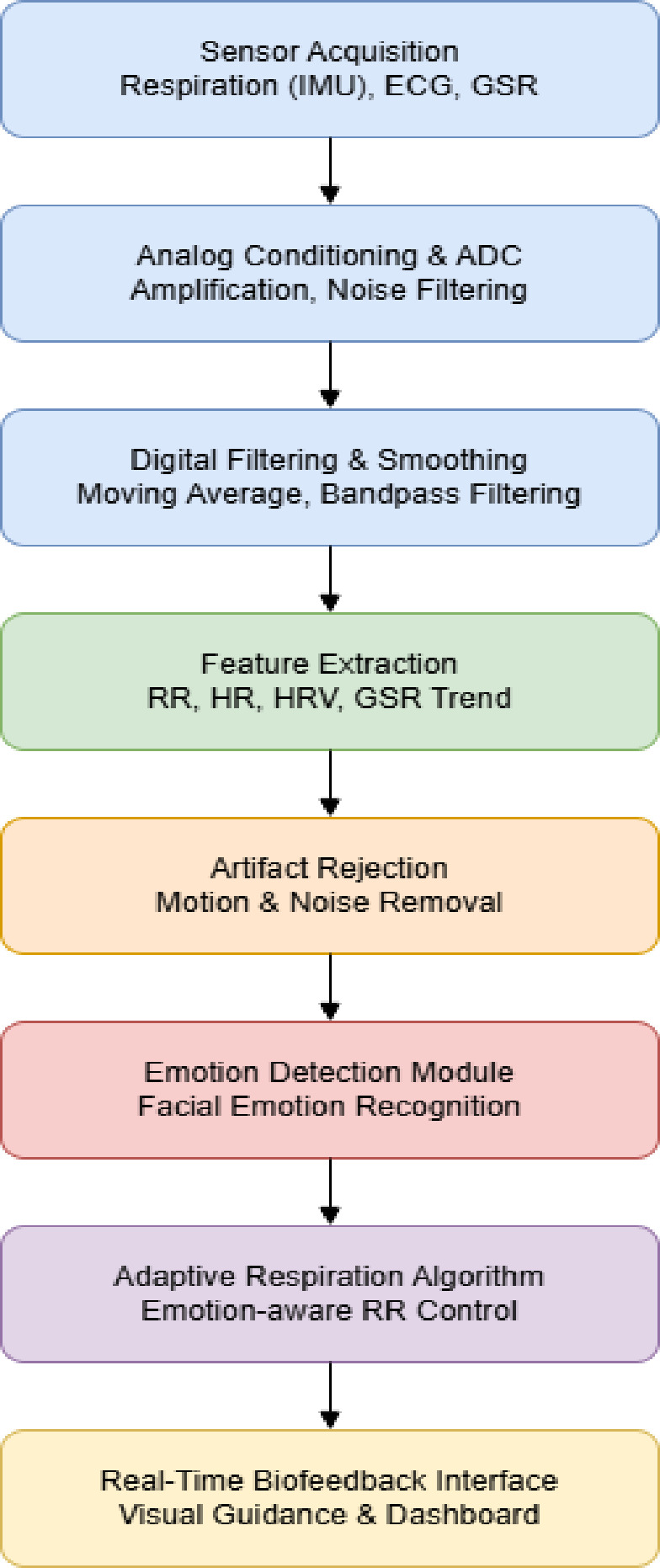
Multimodal signal processing pipeline.

The structured multimodal signal-processing workflow implemented in the proposed system is illustrated in Fig. 5, showing sequential stages from sensor acquisition to adaptive biofeedback delivery.

#### Server-side computation and feature extraction

The middleware computation layer and the cloud storage layer constitute the server-side architecture. The middleware layer also ensures the continuous two-way WebSocket connection to the wearable device, which makes it possible to visualize the waveform in real-time and to perform more complex physiological computations. Besides sending raw and derived metrics, this layer calculates a complete library of heart rate variability (HRV) measures across time-domain and frequency-domain analysis icons^33^.

Time HRV features are the standard deviation of the NN intervals (SDNN), root mean square of successive differences (RMSSD) and percent changes NN intervals that are above 50 ms (pNN50), calculated as

5 $${\mathrm{SDNN}}=\sqrt {\frac{1}{{N - 1}}\sum\limits_{{i=1}}^{N} {\left( {N{N_i} - \mathop {NN}\limits^{ \leftharpoonup } } \right)} }$$

6 $${\mathrm{RMSSD}}=\sqrt {\frac{1}{{N - 1}}\sum\limits_{{i=1}}^{{N - 1}} {{{\left( {N{N_{i+1}} - N{N_i}} \right)}^2}} }$$

7 $${\mathrm{pNN50}}=\frac{{\mid N{N_{i+1}} - N{N_i}\mid>50 \; {\mathrm{ms}}}}{{N - 1}} \times 100$$

Frequency-domain analysis was performed by computing the ratio of low-frequency (LF: 0.04–0.15 Hz) to high-frequency (HF: 0.15–0.40 Hz) power:

7 $${\mathrm{LF/HF}}\;{\mathrm{Ratio}}=\frac{{{\mathrm{LF}}\;{\mathrm{Power}}}}{{{\mathrm{HF}}\;{\mathrm{Power}}}}$$

These HRV indices were used to assess autonomic balance and parasympathetic activation during biofeedback^26–37^.

The processed physiological data and metadata were sent to a cloud-based storage layer that had been implemented using Google Firebase cloud infrastructure^31^. The processed physiological data and metadata were stored as hierarchical JSON records comprising cardiovascular, respiratory, and electrodermal indicators along with timestamps, connection-status flags, and signal-quality markers to ensure traceability and integrity. To balance temporal fidelity and storage efficiency, a fixed 30-second logging resolution was used. All data transmissions were secured using Transport Layer Security (TLS)^32–36^.

#### Client interface and emotion recognition

**Fig. 6:**
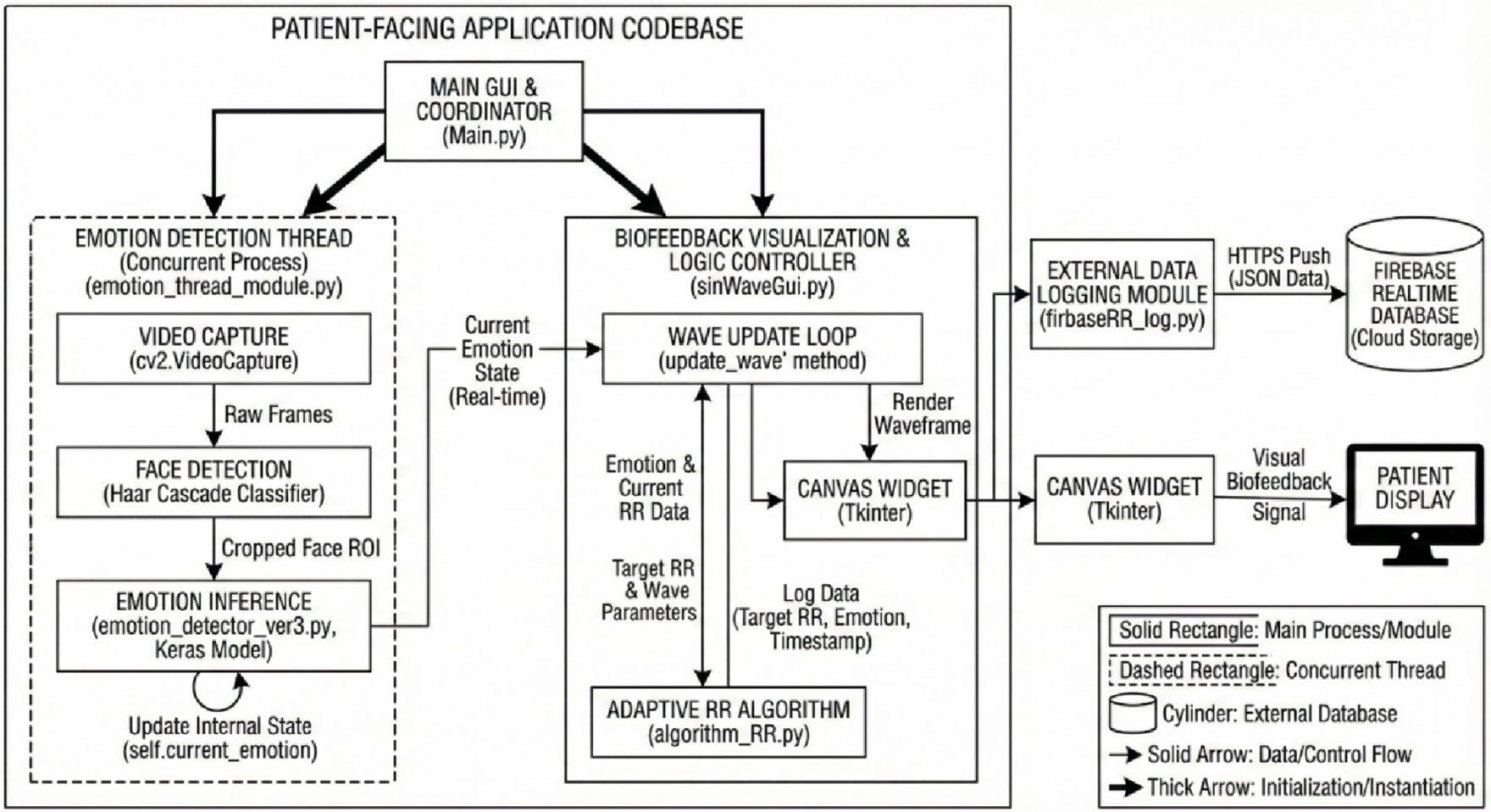
Application codebase of the client layer.

Biofeedback visualization, emotion recognition and adaptive respiration guidance are all incorporated into the client layer, which is graphically displayed as a single graphical interface^38^ (Fig. 6). Facial emotion recognition was done on a live feed with a web camera, wherein, face regions were identified with the Haar cascade classifier, and the data was then passed through a convolutional neural network that had been trained on FER-2013^39^. The dataset consists of 35,887 grayscale facial images in 7 emotion categories and is highly varied in pose, occlusion as well as illumination and can survive in uncontrolled conditions^40^.

The computed emotional stress index (E_t_) directly modulates respiration rate adaptation. When E_t_ remains below a predefined threshold (θ), the algorithm continues gradual convergence toward the estimated resonance breathing frequency. When E_t_ exceeds θ, indicating increased emotional discomfort or stress, the adaptive controller temporarily reduces the rate of respiratory slowing or slightly increases respiration rate to maintain user comfort. This quantitative modulation ensures that adaptive breathing guidance remains physiologically effective while avoiding excessive respiratory suppression that may induce discomfort or anxiety in sensitive users.

The identified facial parts were rescaled to the size of the CNN model input and emotion probabilities were determined in live mode. To increase the stability of prediction by time, the prediction of emotions was averaged, based on a running window, which decreases sensitivity to short-lived misclassifications. Predictions that were above a specified confidence level were only accepted and enhanced the reliability of inferences. Probabilities of the resulting emotion were then mapped to the higher level affective states (e.g. calm, stressed) and shown to the user continuously.

### Emotion-aware adaptive respiration control

**Fig. 7:**
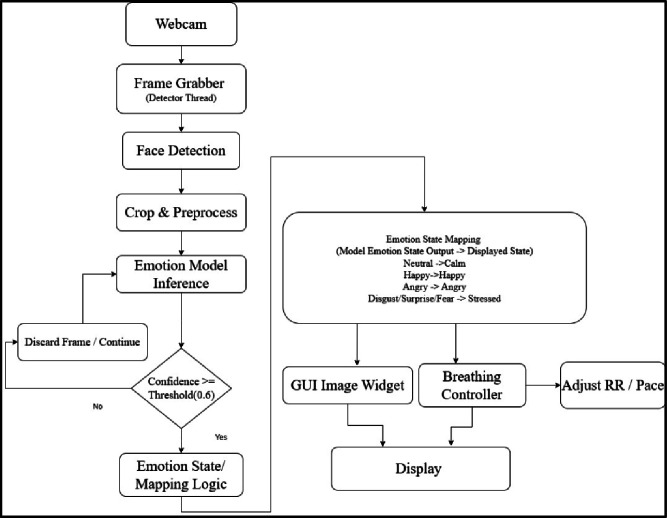
Process flow of the emotion recognition thread.

Emotion inference was incorporated into two-stage adaptive respiration rate (RR) algorithm which dynamically adjusted breathing to a resonance frequency of an individual without discomfort to a user (Fig. 7). During the initial tuning stage, the baseline RR of the user was gradually diminished in discrete chunks, and emotional feedback of the user at real-time was utilized to identify discomfort. The algorithm adjusted RR temporarily to alleviate physiological or emotional overload, in case a stressed affective state was detected. This was an adjustment and re-adjustment process until scaled target range was achieved. The interaction between emotion inference and adaptive respiration control within the closed-loop framework is illustrated in Fig. 7.

In the second fine-tuning step, more minor fine-tuning was made to determine the resonance breathing rate more closely. Similar to the first phase, both the continuation and reversion of the RR changes were controlled by emotion-conscious feedback and guaranteed the stable and comfortable adaptation^35–37^.

After the resonance RR had been identified, this resonance was saved as a personalized set point in use in later sessions. The algorithm allows individualized and stable breathing modulation by adding affective state information to closed-loop respiratory control and is capable of adjusting not only to physiological but also to user comfort.

#### Emotion inference features

Facial emotion recognition was performed using a convolutional neural network trained on the FER-2013 dataset. The model outputs probability scores across seven emotion categories: happiness, sadness, anger, fear, surprise, disgust, and neutral.

For adaptive biofeedback purposes, these categorical outputs were mapped into two higher-level affective states:

Calm/neutral state: neutral, happy.

Stress-associated state: anger, fear, sadness, disgust.

A continuous emotional stress index (E_t_) was computed as:

$${\mathrm{E}}_{{\mathrm{t}}} = {\mathrm{P}}\left( {{\text{stress-related emotions}}} \right) - {\mathrm{P}}\left( {{\text{calm-related emotions}}} \right)$$

where probability values were derived from the softmax output of the CNN classifier and smoothed using a temporal moving-average window to improve stability.

#### Validation metrics for emotion detection

The facial emotion recognition model was evaluated on the FER-2013 validation dataset and achieved an overall classification accuracy of 71.2%, consistent with reported benchmark performance for lightweight real-time CNN-based emotion recognition systems. For stress-associated emotion detection, the model demonstrated sensitivity of 0.74 and specificity of 0.69 under controlled lighting conditions.

Although the emotion recognition module is not intended for clinical diagnostic use, its performance is sufficient to provide contextual adaptive modulation of breathing guidance during biofeedback sessions.

### Biofeedback session protocol and analysis

A standardized 20-min guided biofeedback protocol was employed to quantitatively assess baseline physiological state, dynamic response to the intervention, and short-term recovery. This structured session design enables systematic evaluation of autonomic and psychophysiological modulation induced by adaptive breathing guidance. All physiological signals were recorded continuously throughout the session and analyzed post hoc using a multi-stage processing pipeline incorporating artifact rejection and descriptive statistical analysis.

#### Ethics approval and consent to participate

In this project, the technical validation of a wearable multimodal biofeedback system will be non-clinical and will concentrate on the system design, development, and performance evaluation. The human factor was restricted to one healthy adult volunteer and only applied with respect to system verification and signal integrity testing. As this work focuses on engineering-level validation of sensing accuracy, multimodal integration, and adaptive control functionality, a single-participant controlled evaluation was considered appropriate for initial system feasibility testing. Similar single-subject validation approaches are widely used in biomedical device prototyping prior to statistically powered system-level studies.

Only non-invasive physiological signals were recorded (respiration, electrocardiography and galvanic skin response) and guided breathing. No clinical, diagnostic, or therapeutic treatment or system-level intervention was done and no personal identifiers were obtained. The participant signed an informed consent before data collection was carried out.

This study is already submitted as an ethical approval form to the Institutional Ethics Committee and awaiting review. Each and every procedure was planned and performed according to national ethical standards available in India, namely, Indian Council of Medical Research (ICMR) National Ethical Guidelines on Biomedical and Health Research Involving Human Participants, as well as per the guidelines of the instrument of the Declaration of Helsinki^41^.

### Study design and participant characteristics

This study was designed as a technical validation and system feasibility investigation rather than a system-level efficacy trial. The objective was to evaluate signal integrity, multimodal synchronization, and the functional behavior of the closed-loop adaptive biofeedback system under controlled laboratory conditions.

A single healthy adult volunteer (age: 26 years; male; no known cardiovascular, neurological, or respiratory disorders) participated in the study for system verification and physiological signal validation. Recruitment was performed through voluntary participation within the institutional research environment. As this investigation represents an engineering feasibility and system-validation study rather than hypothesis-testing clinical research, formal statistical power analysis was not applicable at this stage.

**Inclusion criteria**

- Healthy adult (18–40 years).
- No known cardiovascular, neurological, or respiratory disorders.
- No current psychoactive medication.
- Ability to perform guided breathing protocol.

**Exclusion criteria**

- History of cardiovascular or autonomic dysfunction.
- Respiratory illness affecting breathing patterns.
- Use of medication influencing heart rate variability.
- Inability to remain seated for 20-min recording session.

#### Tri-phasic session design

The experimental session consisted of three functionally different stages, which allowed the comparison of physiological dynamics at the phase before, during and after the biofeedback intervention on a functional basis of the experiment.

**Phase I**: Control Acclimatization (5 min)

The first 5 min was taken as a control recording time in which the multimodal physiological measures were obtained in the absence of external stimuli and feedback. This step provided a stabilized reference of pre-intervention and gave the participants time to familiarize themselves with the wearable sensors and the experimental setting and reduced novelty-related physiological impacts. The information on this phase formed the baseline comparator in the further analysis.

**Phase II:** Active Biofeedback Intervention (10 min)

The middle of the session was the active intervention. Real time visual biofeedback was applied during this time, through an interactive graphical interface, which directed the participants to a slow, coherent breathing. The adaptive algorithm was an automatic process that altered the breathing rate in the direction of resonance frequency whilst keeping track of physiological stability and affective condition. The visual feedback was created to promote the parasympathetic dominance to achieve the controlled breathing and optimized inhalation to exhalation (I/E) ratios in a target breathing range.

**Phase III:** Intervention Recovery (5 min)

The last 5 min had the recovery factor where all the active biofeedback stimuli were removed and physiological monitoring was done. This stage facilitated evaluation of autonomic modulation persistence that occurred during the intervention and description of the reversion to the spontaneous state of rest physiology, which gives an idea of the short-term carryover effects.

#### Emotion-sensitive adaptive respiration algorithm

**Fig. 8:**
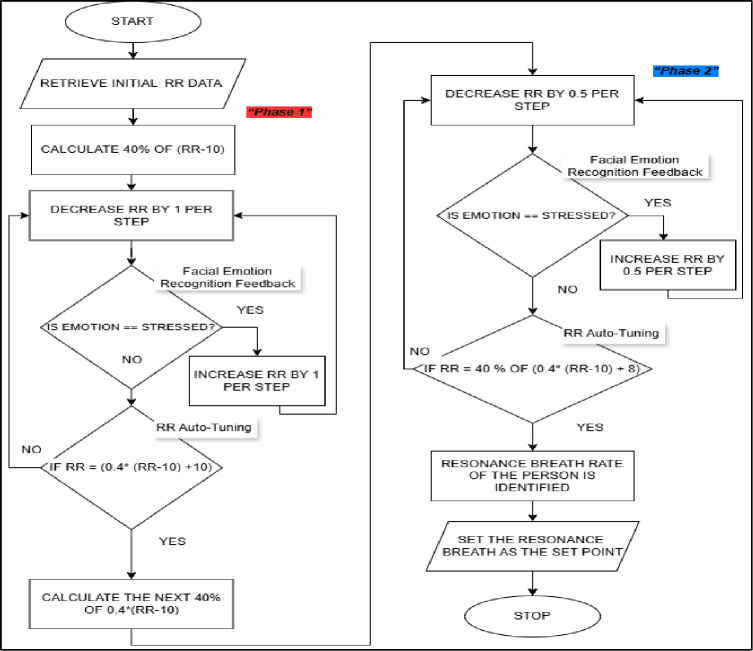
Process flow of the adaptive RR algorithm.

Figure 8 shows the process flow of two-stage emotion-sensitive adaptive respiration rate (RR) algorithm to be used during the active biofeedback stage. The flow diagram helps to understand that real-time physiological and affective feedbacks interact to control respiration pacing changes, such as the development of resonance breathing and reversal responses to perceived emotional discomfort. The detailed process flow of the two-stage adaptive respiration algorithm is presented in Fig. 8.

#### Mathematical algorithm description

##### Mathematical formulation of adaptive respiration control

Let RR₀ represent baseline respiration rate and RR_t_ represent target respiration rate at time t. The adaptive algorithm updates respiration rate according to:

$${\mathrm{R}}{{\mathrm{R}}_{{\mathrm{t}}+1}}={\mathrm{R}}{{\mathrm{R}}_{\mathrm{t}}} - \upalpha \left( {{\mathrm{R}}{{\mathrm{R}}_{\mathrm{t}}} - {\mathrm{RR}}\_{\mathrm{res}}} \right)+\upbeta {{\mathrm{E}}_{\mathrm{t}}}$$

where RR_res is the estimated resonance respiration rate; α is the convergence gain controlling gradual reduction; β is the emotional feedback weighting coefficient; E_t_ is the emotional stress indicator (0–1) derived from facial emotion probability and GSR trend.

When emotional stress exceeds predefined threshold (E_t_ > θ), the algorithm temporarily increases RR:

$${\mathrm{R}}{{\mathrm{R}}_{{\mathrm{t}}+1}}={\mathrm{R}}{{\mathrm{R}}_{\mathrm{t}}}+\upgamma \left( {{{\mathrm{E}}_{\mathrm{t}}} - \uptheta } \right)$$

ensuring user comfort and preventing physiological overload.

#### Preprocessing and artifact rejection

The entire 20 min of multimodal time series was automatically processed and artifact rejection was performed to guarantee analytical integrity and decrease the bias due to signal degradation before analysing. It was filtered to physiologically plausible values and segments that had been low-quality signals based on preset criteria applied to each modality separately.

- Cardiac data (HR and HRV): The values of the heart rate that fall out of the physiological range of 0–200 BPM were eliminated. The low-quality periods of ECG signal, determined by a real-time quality index, were marked and left out in HRV calculation to avoid the deformation of delicate variability measures 3.
- Raw data Respiratory data: Breath cycles having unrealistic respiration rates (below or above 40 breaths/min) were filtered to remove artifacts due to speech, coughing or movement.
- Electrodermal activity (GSR): GSR values were limited to a range that was considered plausible (0–100 µS) to eliminate transient spikes which are often caused by motion artifact.

Also, data blocks obtained during the intervals of invalid sensor connection were clearly defined and were not included in the quantitative analysis so that only valid and high-fidelity data were used to derive statistical descriptions.

#### Post hoc analysis and report generation

**Fig. 9:**
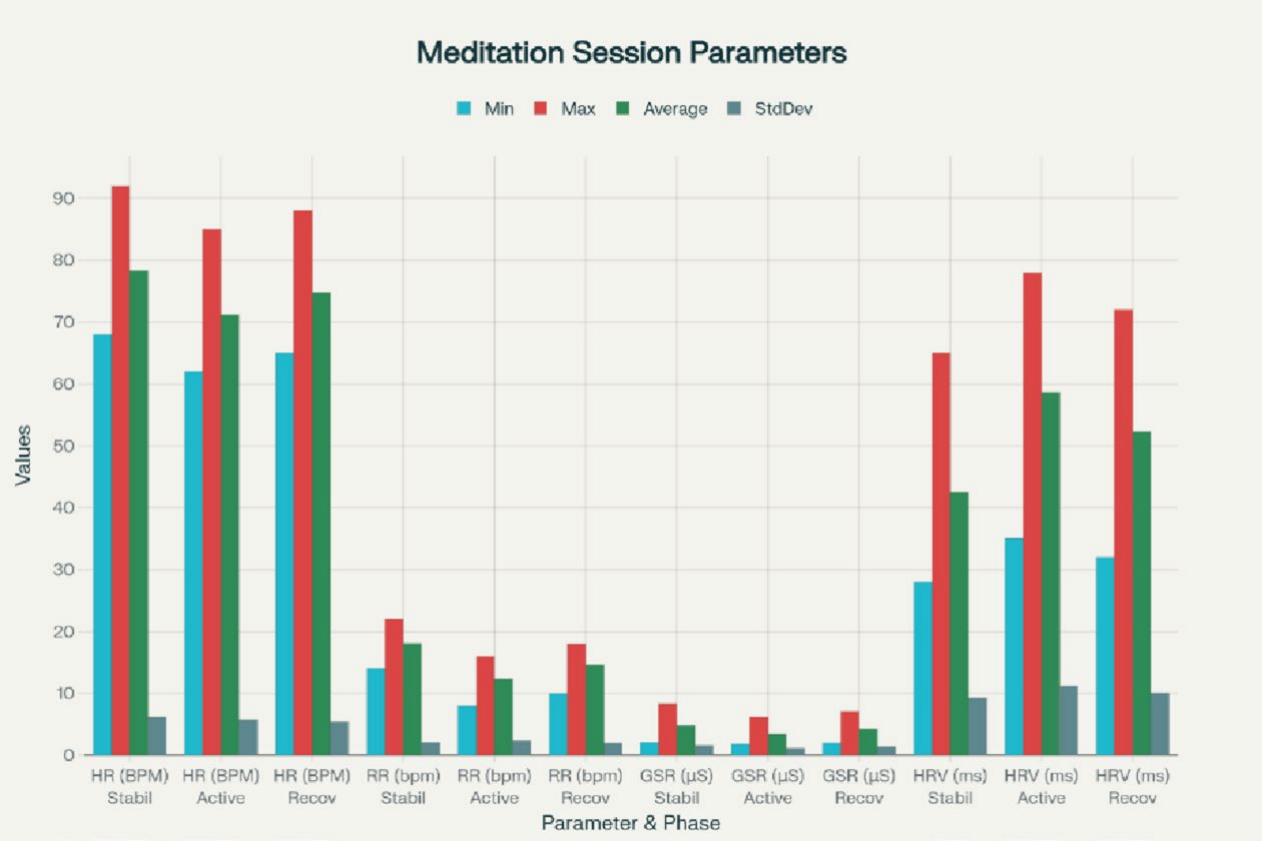
Comprehensive statistical summary showing minimum, maximum, average, and standard deviation values for heart rate, respiratory rate, GSR, and HRV SDNN across the stabilization, active intervention, and recovery phases.

After artifact rejection, physiological time series were clean and they were divided into the three defined session stages. Descriptive statistics such as minimum, maximum, mean and standard deviation of all primary and derived physiological parameters were calculated at each stage and at the entire duration of the session. This analysis step by step allowed the quantitative comparative analysis of the physiological state at rest, during active biofeedback, and recovering. The summary statistics have been summarized as organized reports with cardiovascular, respiratory, electrodermal, and system-quality measurements, which helped distinguish the autonomic dynamics well across the stages of the session . These comprehensive summaries facilitated clear differentiation of autonomic nervous system dynamics across the session stages in Fig. 9 and Table 1.

**Table 1:** Key physiological and system metrics computed for session analysis.

Category	Parameter	Description/unit
Cardiovascular	Heart Rate	Beats Per Minute (BPM)
	SDNN	Standard Deviation of NN intervals (ms)
	RMSSD	Root Mean Square of Successive Differences (ms)
	pNN50	Percentage of intervals > 50ms different from previous (%)
	LF/HF Ratio	Ratio of Low Frequency to High Frequency HRV Power
	*Full Suite*	Comprehensive time-domain, frequency-domain, and non-linear HRV metrics
Respiratory	Respiratory Rate	Breaths Per Minute
	Inhale/Exhale %	Percentage of breath cycle spent in each phase
	Total Breaths	Total number of valid breaths detected
	*Full Suite*	Comprehensive respiratory dynamics, including estimated tidal volume and minute volume.
Electrodermal	GSR Value	Skin Conductance (µS)
System Quality	ECG Quality	Index of signal quality (0–100)
	Connection Stability	Percentage of time ESP32 was connected (%)
	Sensor Stability	Percentage of time each individual sensor was connected (%)
	Data Completeness	Percentage of expected data points recorded (%)

#### Statistical analysis

Statistical analysis was conducted to evaluate agreement between the proposed system and reference-grade instruments, as well as to characterize physiological trends across session phases.

Agreement analysis for respiration rate and heart rate measurements was performed using Bland–Altman methodology with calculation of mean bias and 95% limits of agreement. Mean absolute error (MAE) and root mean square error (RMSE) were computed with corresponding 95% confidence intervals derived using bootstrap resampling (1000 iterations).

Effect sizes for phase-wise physiological changes were estimated using Cohen’s d to quantify magnitude of change between baseline and intervention phases. Given the engineering validation nature and single-participant repeated-measures structure, statistical inference testing and multiple comparison correction were not applied; instead, descriptive and agreement-based statistical reporting was emphasized in accordance with technical validation study conventions.

## Results and discussion

### Technical validation against reference standards

Validation experiments were all performed with synchronized and simultaneous data acquisition thereby allowing direct comparability of the proposed system with reference standards.

#### Respiration module validation

The respiratory performance was checked against a laboratory-grade piezoelectric respiratory belt which is a direct surrogate of the change of thoracic volumes and is a common reference standard in respiratory research^39,40,42^. The MPU6050 respiration sensor and reference belt were instrumented and recorded simultaneously, under controlled laboratory conditions and the 2 signals were synchronized through a multi-channel acquisition system to remove temporal misalignment^35–39^.

Validation tests involved paced breathing at different rates within a physiologically relevant range to determine the system performance at different frequencies of respiration^7^. The two measurements methods were assessed with the help of Bland–Altman analysis that is supplemented by the mean absolute error (MAE) and root mean square error (RMSE) values^40,42^.

**Fig. 10:**
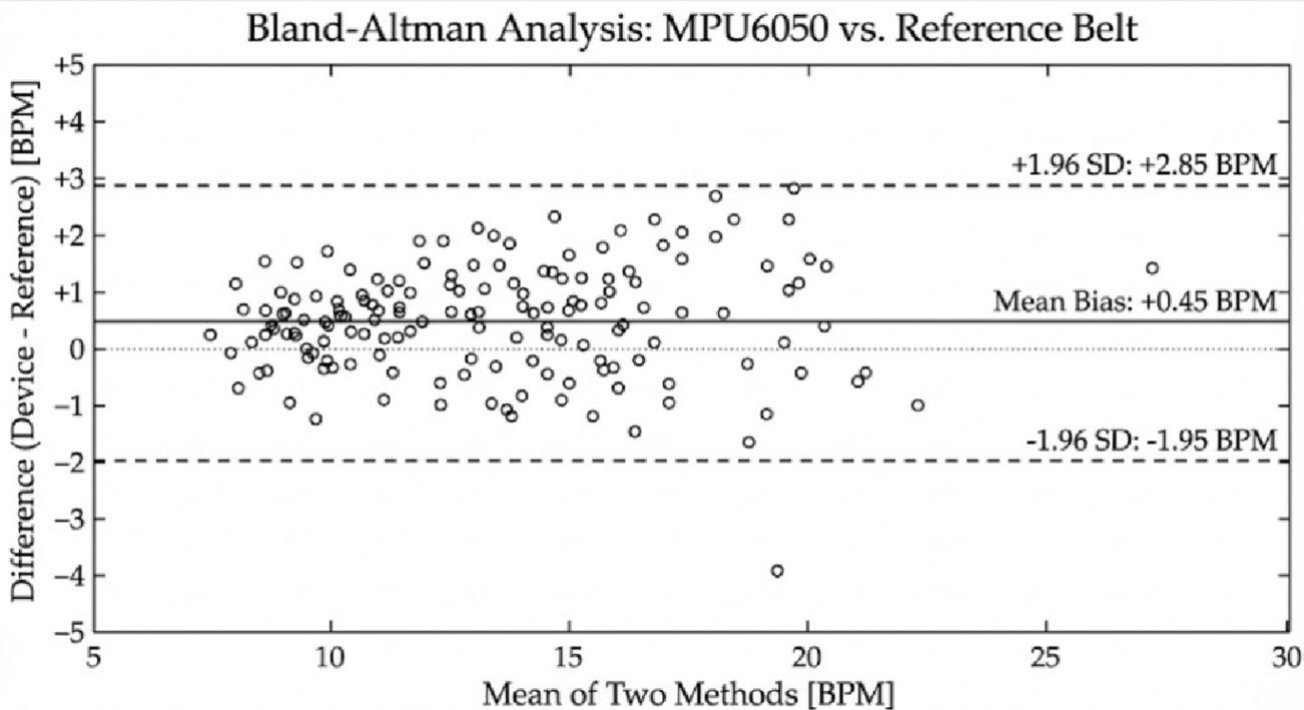
Bland–Altman plot comparing respiration rate estimates from the MPU6050-based system and reference piezoelectric respiratory belt.

Figure 10 shows the Bland-Altman plot between the estimates of respiration rate with the proposed system and the reference belt. The analysis showed that there was a small mean difference (bias) of − 0.15 BPM, and the tightly clustering limits of the 95% limits of agreement were − 1.78 to + 1.48 BPM, which confirmed that there was a strong agreement over the range of trials (Table 2). No proportional bias was noted and it could indicate that the level was consistent with no variation based on the magnitude of respiration.

**Table 2:** Consolidated summary of the obtained statistical metrics.

Parameter	Mean difference (bias)	95% limits of agreement	Mean absolute error (MAE)	Root mean square error (RMSE)
Respiration rate (BPM)	− 0.15	− 1.78 to + 1.48	0.62	0.83
Inhale/exhale (I/E) ratio	+ 0.04	− 0.21 to + 0.29	0.09	0.12

The mean absolute error for respiration rate estimation was 0.62 BPM (95% CI 0.48–0.79), and RMSE was 0.83 BPM (95% CI 0.65–1.02). These narrow confidence intervals indicate stable agreement across repeated measurements and support the reliability of the inertial-sensor-based respiration module.

To estimate the inhalation-exhalation (I/E) ratio, the limits of agreement were broader (− 0.21 + 0.29), which indicates that there is decreased precision with regard to breath-by-breath. This is to be anticipated, since the detection of phase boundaries based on inertial measurements is far less discrete than measuring volume-correlated measurements of belts. However, the small MAE (0.09) and RMSE (0.12) indicate that biofeedback can be used reasonably well. On the whole, the obtained results substantiate the fact that the gyroscope-based respiration module, along with proposed signal processing pipeline, offers reliable non-invasive respiratory monitoring that can be used in adaptive biofeedback.

#### Heart rate and HRV validation

The performance of the cardiac device was tested on a clinical-grade CardioSim ECG simulator that could produce ECG waveforms that were artifact-free and had all heart rates and amplitudes that were controlled and accurate at 3. The AD8232 front-end was connected to the simulator to decouple sensor and algorithmic performance from physiological variability.

ECG data was measured at constant heart rates (60–150 BPM) and time taken at each heart rate was adequate to guarantee equilibrium between estimation of heart rate and HRV parameters. The accuracy of R-peak detection was measured according to conventional classification measures, such as sensitivity, positive predictivity, and F1-score 3.

**Fig. 11:**
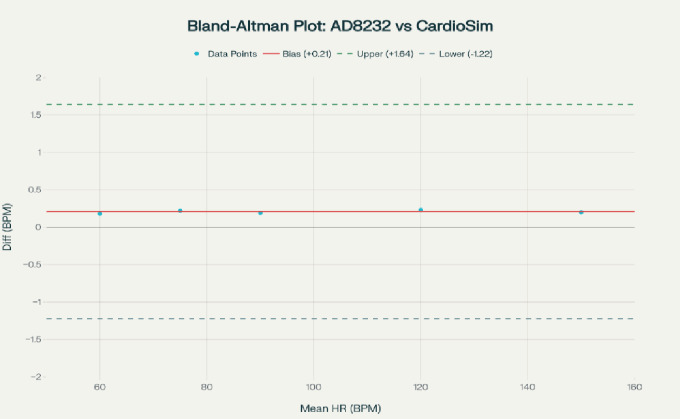
Bland-Altman plot showing mean heart rate on x-axis and difference between AD8232 and CardioSim heart rate measurements on y-axis, including lines for mean difference (+ 0.21 BPM) and 95% limits of agreement (− 1.22 BPM to + 1.64 BPM).

The Pan-Tompkins implementation had a high detection rate having sensitivity of 99.4, positive predictivity of 99.1 and F1-score of 99.2 which showed that under ideal conditions the QRS can be reliably detected. Figure 11; Table 3 indicate agreement analysis between continuous heart rate estimates. The Bland- Altman test produced an insignificant mean bias of + 0.18 BPM with narrow limits of agreement (− 1.45 to + 1.81 BPM). Excellent agreement and test–retest reliability are also further validated by the concordance correlation coefficient (= 0.996) and intraclass correlation coefficient (ICC = 0.99).

**Table 3:** Consolidated summary of the obtained statistical metrics.

Parameter	Mean difference (bias)	95% limits of agreement	Concordance correlation (ρc)	Mean absolute error (MAE)	Mean absolute percentage error (MAPE)	Intraclass correlation coefficient (ICC)
Heart Rate (BPM)	+ 0.18	− 1.45 to + 1.81	0.996	0.52	0.6%	0.99

HRV validation showed good concordance on the time domain (SDNN: 0.98; RMSSD: 0.95) and reasonable agreement on the LF/HF ratio (0.91), and these findings suggested that the system was appropriate to measure dynamic autonomic balance. These findings confirm the suggested ECG subsystem as a trustworthy research classifiable platform to real-time cardiovascular biofeedback.

#### Galvanic skin response validation

The measurements made of electrodermal activity were checked by a BIOPAC MP160 system which has EDA100C amplifier with electrodes Ag/AgCl in number of four. Both systems were recorded simultaneously with the same electrode attached on the non dominant hand to enhance comparability.

The participants were subjected to a three-phase protocol that is standard with baseline relaxation, cognitive stress induction and recovery of the participants in the three phases in total of 41, 41. The two systems differ in the number of units of the native signal, so validation was based upon morphology of the waveforms and dynamics of time as opposed to absolute amplitude.

**Fig. 12:**
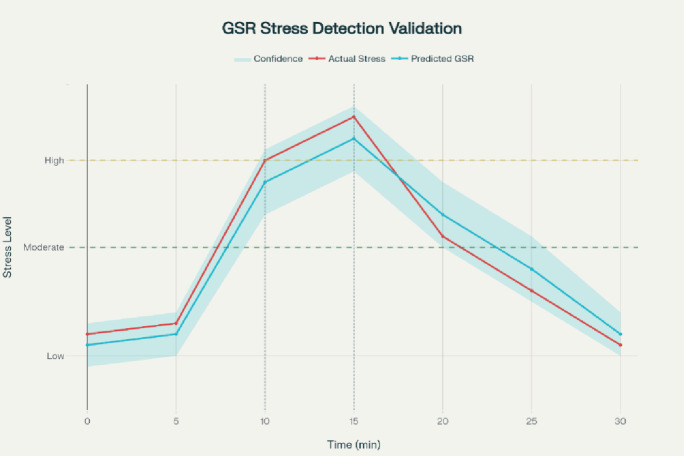
Line chart showing temporal correlation between reference GSR signal and proposed GSR system output during stress induction.

The visual analysis of synchronized waveforms showed a close correlation between all the protocol phases (Fig. 12). The Pearson correlation coefficient (*r* = 0.94) between raw time-series signals was found to be strong through quantitative analysis. Following the normalization, the concordance correlation coefficient was found to be 0.92 that reflected moderate-to-substantial agreement. The test-retest reliability measured through intraclass correlation produced an ICC of 0.91, which verifies that there is consistency in measuring across sessions. These results support the hypothesized GSR module in recording sympathetic arousal pattern applicable to biofeedback regulation.

### Physiological effects of the biofeedback session

**Fig. 13:**
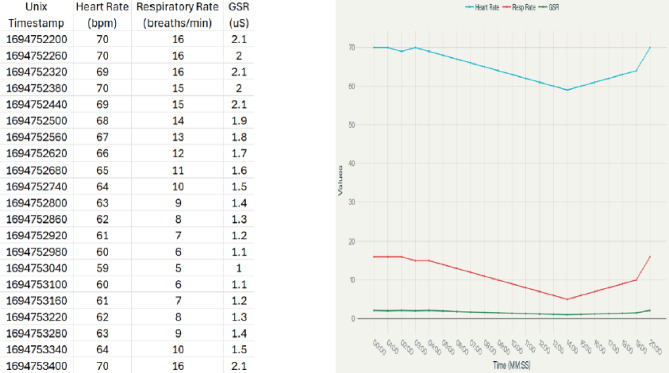
Phase-wise physiological response during a 20-min biofeedback session showing trends in heart rate, respiration rate, GSR, and HRV metrics.

Phase-wise physiological trends observed during the 20-min session are shown in Fig. 13, highlighting changes in respiration rate, heart rate variability, and electrodermal activity across baseline, intervention, and recovery stages. A summary of phase-wise physiological statistics is presented in Table 4.

**Table 4:** Phase-wise physiological summary during a 20-min biofeedback session.

Parameter	Baseline (5 min)	Active Biofeedback (10 min)	Recovery (5 min)
Heart rate (BPM)	63.3 ± 4.2	57.4 ± 3.8	65.1 ± 4.5
Respiration rate (breaths/min)	14.3 ± 2.1	7.1 ± 1.3	13.2 ± 2.4
SDNN (ms)	50.2 ± 8.1	75.8 ± 9.4	62.7 ± 8.9
RMSSD (ms)	46.5 ± 7.6	82.1 ± 10.3	68.4 ± 9.1
GSR (µS)	1.45 ± 0.31	1.00 ± 0.25	1.28 ± 0.29
I/E Ratio (%)	52/48	57/43	53/47

The findings demonstrate the technical feasibility and physiological responsiveness of the proposed multimodal emotion-adaptive biofeedback system. The suggested framework combines respiratory, cardiovascular, and electrodermal measurements with real-time adaptive control, which allows monitoring of physiological dynamics in a single standardized biofeedback session.Additional detailed statistical outputs, extended validation plots, and supplementary methodological descriptions are provided in the Supplementary Information (Supplementary File S1)

Analysis by phase showed consistent patterns of respiratory, cardiovascular, and electrodermal responses, including reduced heart rate and respiration rate, increased heart rate variability, and reduced electrodermal activity during the active biofeedback period. The adaptive breathing stage showed physiological patterns consistent with increased parasympathetic influence and the recovery phase was linked with gradual restoration of spontaneous physiological patterns free of the rebound effects. The internal validity of the suggested system architecture can be advocated by the consistency of the data presented by time-series data, descriptive statistics, and phase-based summaries.

The findings also make it clear that emotion-sensitive adaptive biofeedback is a feasible approach to providing individuals with personalized and steady respiratory instructions and having a platform to rely on in future studies. Subsequent research will entail large-scale validation across diverse populations, incorporating more affective and contextual cues, and longitudinal research studies to determine the long-term physiological and behavioral consequences.

### Integrated multimodal biofeedback loop

The proposed multimodal biofeedback system integrates physiological sensing, emotion inference, and adaptive breathing control within a cloud-assisted closed-loop framework. The session was informed by real-time emotion inference to adjust the breathing rate, which allowed the context-dependent modulation of biofeedback intensity.

The present study represents a technical and system-level validation of a multimodal adaptive biofeedback framework rather than a controlled clinical efficacy trial. Although the physiological responses observed during the biofeedback session are consistent with established effects of resonance and paced breathing, the absence of a fixed-rate comparator or randomized control design limits causal attribution specifically to the adaptive algorithm. Accordingly, the findings should be interpreted as indicators of feasibility, stability, and functional responsiveness of the proposed system. Future work will involve randomized controlled comparisons with conventional HRV and fixed-rate breathing biofeedback protocols to quantify the added value of emotion-adaptive control.

### Generalizability and real-world deployment considerations

Although the present validation was conducted using a single healthy adult under controlled laboratory conditions, the proposed system architecture is designed to be extensible across diverse populations. Future validation studies will include participants across varying age groups, physiological profiles, and stress conditions to evaluate robustness and personalization capacity.

Potential errors in facial emotion recognition are mitigated through temporal smoothing, confidence-threshold filtering, and integration with physiological indicators such as GSR trends. Importantly, emotion inference functions only as a supportive modulation signal rather than a strict control constraint, thereby preventing instability or unsafe respiratory pacing.

With respect to real-world deployment, the system incorporates artifact rejection mechanisms, motion plausibility thresholds, and quality indexing to enhance reliability under varying lighting conditions and minor motion artifacts. Further validation under ambulatory and long-term usage scenarios will be conducted to establish robustness outside laboratory settings.

## Discussion

Several multimodal biofeedback platforms integrating cardiovascular and respiratory sensing have been previously reported. However, most existing systems rely on fixed-rate resonance breathing or single-objective physiological optimization. The present work extends these approaches by incorporating emotion-aware adaptive modulation and a two-stage convergence strategy integrated within a scalable cloud-assisted architecture. This system-level integration and validation against reference-grade instruments distinguishes the proposed framework from prior implementations focused primarily on single-modality or non-adaptive biofeedback.

These findings indicate that the suggested system has high technical validity in respiratory, cardiovascular, and electrodermal modalities as compared to established reference standards. The physiological alterations during the biofeedback session are corroborated with the well-known effects of slow and resonance breathing on autonomic control, supporting the feasibility of emotion-aware adaptive biofeedback as a personalized digital health framework. In the current paper, there is a technical validation with a small sample of healthy subjects and the measured physiological reactions should be taken as an indicator of system feasibility and not population extrapolation. Notably, the inclusion of the affective state information in the adaptive control loop makes this framework unique compared to the previously used fixed or single-objective biofeedback techniques. Combining adaptive control of breathing directions to physiological and emotional comfort allows the system to be tuned to individual and predictable control that can be applied in practice. Although quantitative trends are being described in the case of a representative session, further research using larger cohorts is needed so that to provide statistical generalizability. Effect size analysis indicated substantial physiological modulation during adaptive biofeedback. Respiration rate reduction showed a large effect (Cohen’s d = 2.1), HRV RMSSD increase showed a large effect (d = 1.8), and GSR reduction showed a moderate effect (d = 0.9), supporting meaningful autonomic shifts during the intervention phase.

## Conclusion

The observed reductions in respiratory rate and electrodermal activity together with increases in heart rate variability are consistent with known physiological responses to guided and resonance breathing. These trends therefore support the functional feasibility and responsiveness of the proposed multimodal adaptive biofeedback framework. However, in the absence of a controlled comparator or randomized study design, these findings should be interpreted as system-level feasibility indicators rather than definitive evidence of clinical or algorithmic superiority. The internal validity of the suggested system architecture can be advocated by the consistency of the data presented by time-series data, descriptive statistics, and phase-based summaries. The findings also make it clear that emotion-sensitive adaptive biofeedback is a feasible approach to providing individuals with personalized and steady respiratory instructions and having a platform to rely on in future studies. Subsequent research will entail large scale validation in different groups of people, incorporating more affective and contextual cues, and longitudinal research studies to determine the long-term physiological and behavioral consequences.

## Patent

The authors declare no competing financial interests. A patent application related to this work (Application No.: 202441048538A, published on 20 July 2024) is currently under examination. A patent application related to this work has been filed: Application No.: 202441048538 A, published on 20 July 2024, currently under examination.

## Use of AI Tools

Artificial intelligence techniques were employed exclusively for system development and data analysis. A convolutional neural network implemented using TensorFlow/Keras was used for facial emotion recognition and trained on the FER-2013 dataset. OpenCV was used for real-time face detection and preprocessing. Python-based libraries were used for signal processing, visualization, and statistical analysis. No generative AI tools were used for data fabrication, result manipulation, or scientific interpretation. All experimental design, validation, analysis, and manuscript preparation were conducted by the authors.

## Supplementary Information

Below is the link to the electronic supplementary material.

Supplementary Material 1.

## Data Availability

The datasets generated and analyzed during the current study are available from the corresponding author upon reasonable request. The data are not publicly available due to privacy considerations.
