# Supplementary material for: Technical validation of a multimodal emotion-adaptive biofeedback system for autonomic regulation using guided breathing
Source: Sci Rep. 2026 Apr 1;16:15327. doi: 10.1038/s41598-026-46105-9 (PMC13181121; doi:10.1038/s41598-026-46105-9)
Supplement: Supplementary file 1 — Supplementary Material 1. [file 41598_2026_46105_MOESM1_ESM.docx]

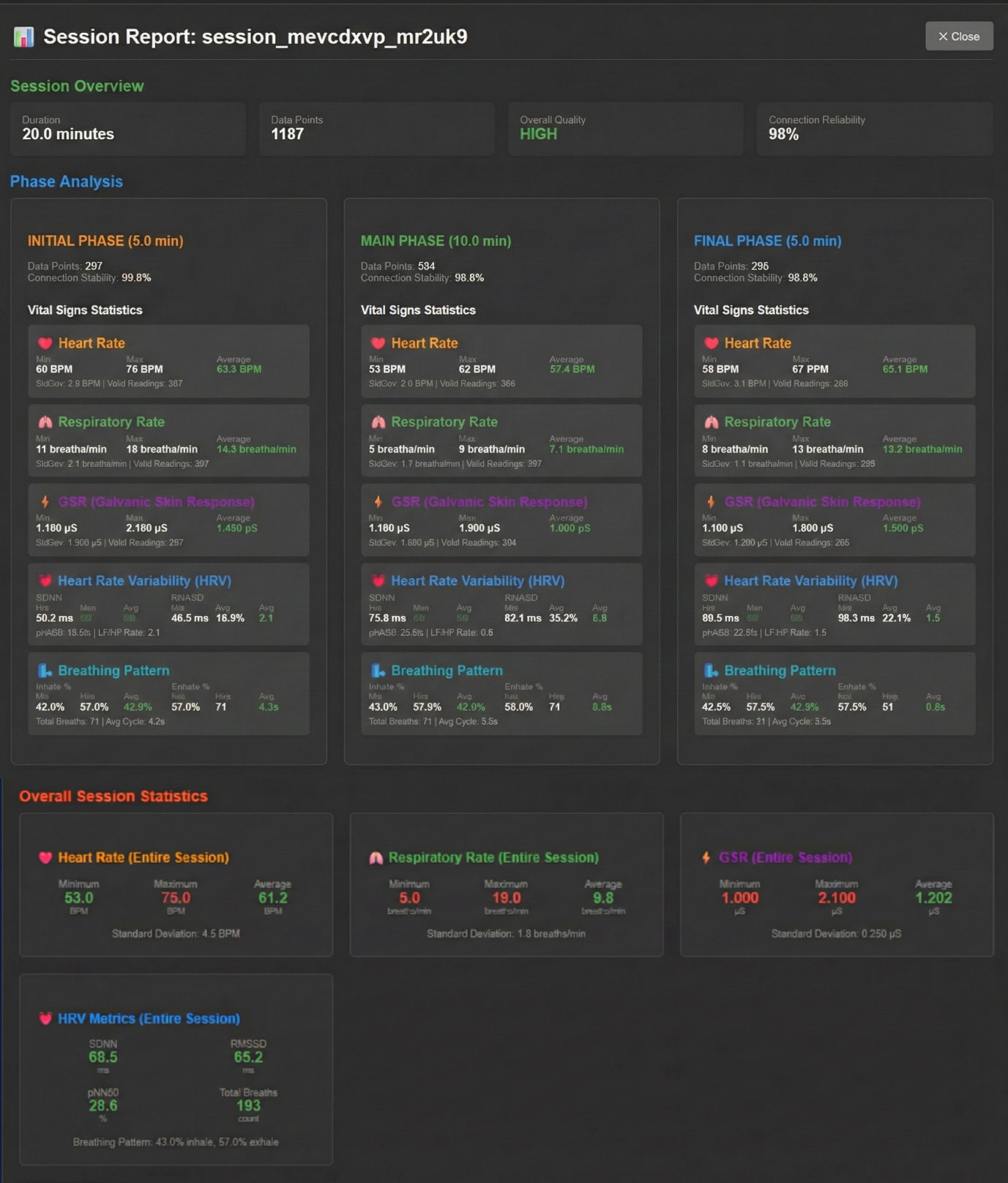


**Fig 14** - Phase-wise physiological response during a 20-minute biofeedback session showing trends in heart rate, respiration rate, GSR, and HRV metrics.
